# Colistin–niclosamide-loaded nanoemulsions and nanoemulsion gels for effective therapy of colistin-resistant *Salmonella* infections

**DOI:** 10.3389/fvets.2024.1492543

**Published:** 2024-10-23

**Authors:** Junkai Zhang, Xilong Wang, Pengliang Li, Yanling Gao, Ruiyun Wang, Shuaihua Li, Kaifang Yi, Xiaodie Cui, Gongzheng Hu, Yajun Zhai

**Affiliations:** ^1^College of Veterinary Medicine, Henan Agricultural University, Zhengzhou, China; ^2^Henan Vocational College of Agriculture, Zhengzhou, China

**Keywords:** colistin, niclosamide, bacterial resistance, *Salmonella*, nanoemulsions, nanoemulsion gels

## Abstract

Colistin (COL) is regarded as a last-resort treatment for infections by multidrug-resistant (MDR) Gram-negative bacteria. The emergence of colistin-resistant Enterobacterales poses a significant global public health concern. Our study discovered that niclosamide (NIC) reverses COL resistance in *Salmonella* via a checkerboard assay. However, poor solubility and bioavailability of NIC pose challenges. In this study, we prepared a self-nanoemulsifying drug delivery system (SNEDDS) co-encapsulating NIC and COL. We characterized the physicochemical properties of the resulting colistin–niclosamide-loaded nanoemulsions (COL/NIC-NEs) and colistin–niclosamide-loaded nanoemulsion gels (COL/NIC-NEGs), assessing their antibacterial efficacy *in vitro and in vivo*. The COL/NIC-NEs exhibited a droplet size of 19.86 nm with a zeta potential of −1.25 mV. COL/NIC-NEs have excellent stability, significantly enhancing the solubility of NIC while also demonstrating a pronounced sustained-release effect. Antimicrobial assays revealed that the MIC of COL in COL/NIC-NEs was reduced by 16–128 times compared to free COL. Killing kinetics and scanning electron microscopy confirmed enhanced antibacterial activity. Antibacterial mechanism studies reveal that the COL/NIC-NEs and COL/NIC-NEGs could enhance the bactericidal activity by damaging cell membranes, disrupting proton motive force (PMF), inhibiting multidrug efflux pump, and promoting oxidative damage. The therapeutic efficacy of the COL/NIC-NEs and COL/NIC-NEGs is further demonstrated in mouse intraperitoneal infection models with COL-resistant *Salmonella*. To sum up, COL/NIC-NEs and COL/NIC-NEGs are a potentially effective strategies promising against COL-resistant *Salmonella* infections.

## 1 Introduction

Currently, the emergence and prevalence of multidrug-resistant (MDR) and pandrug-resistant (PDR) Gram-negative bacteria pose significant challenges to the treatment of clinical infections (1–3). The emergence and spread of MDR Gram-negative bacteria represent a threat to human and animal health (4). *Salmonella* is an important zoonotic pathogen with the highest incidence of *Salmonella* infection, causing considerable economic losses due to the culling and replacing of infected flocks and unbearably high treatment costs to poultry farmers (5, 6). Colistin (COL), a non-ribosomal peptide antibiotic, is often regarded as a last-resort option against MDR Gram-negative bacteria. However, the appearance of *mcr-1* and its mutants seriously threatens the clinical therapeutic efficacy of colistin (7, 8). The widespread dispersion of plasmid-mediated *mcr* resistance gene in swine, poultry, cattle, and sheep poses a challenge to the last line of defense, which could pose a serious threat to public health (9–11). Therefore, there is an urgent need to discover new and effective antimicrobial therapeutic strategies.

Combining existing antibiotics with adjuvants offers a promising strategy for the effectiveness in treating MDR bacterial infections (12). There are many kinds of COL adjuvants reported, for example, natural extract nordihydroguaiaretic acid and tetrandrine (13, 14), Gram-positive antibiotics clarithromycin and rifampicin (15), and anti-parasitical medicine oxyclozanide and closantel (16, 17). Despite these ongoing efforts, no COL adjuvant has been applied in human medicine or veterinary clinical practice so far, due to practical and technical limitations.

Niclosamide (NIC) is an FDA-approved anthelmintic drug widely used for treating tapeworm infection in humans and has lately been shown to treat viral diseases such as COVID-19 and possess anti-diabetic activities (18, 19). In addition, a previous study demonstrated that NIC can reverse COL resistance in *Acinetobacter baumannii* and *Klebsiella pneumoniae* (17, 20, 21). However, NIC belongs to class II, according to the Biopharmaceutics Classification System (BCS), which is indicative of poor aqueous solubility, low bioavailability, and dissolution rate-limited absorption (22). Due to this limitation, its combination with COL directly affects the clinical therapeutic effect. Therefore, there is an urgent need to develop a drug delivery system capable of encapsulating both hydrophilic COL and hydrophobic NIC to achieve synergistic antibacterial activity.

Nanotechnology is an emerging and rapidly evolving technology that offers possibilities for diverse applications (23). Nanoemulsions (NEs), a droplet size of 10 to 100 nm, are generally made up of an oil, surfactant, co-surfactant, and water (24). These nanoemulsion systems offer prospective carriers for improving the dissolution of poorly soluble drugs that have a higher drug load capacity, better visual transparency, and better performance (24, 25). Higher stability and better solubility of solutes are also characteristics of nanoemulsions, and the drugs may be loaded in the internal phase or distributed in the external phase of nanoemulsions (26). Nanoemulsion gel is a drug delivery system that combines nanoemulsions with gel matrices to create a sustained-release function. Nanoemulsions and nanoemulsion gels are two types of nanocarriers that have been shown to enhance drug efficacy (27).

In this study, the nanoemulsions and nanoemulsion gels co-loaded with NIC and COL were prepared to reverse COL resistance. First, we evaluated the synergistic antibacterial activity of NIC and COL. Then, we developed a self-nanoemulsifying drug delivery system (SNEDDS) to serve as a vehicle for delivering COL and NIC concurrently. The particle size, stability, and release characteristics of COL/NIC-NEs and COL/NIC-NEGs were thoroughly evaluated, while their antibacterial activity and underlying mechanism were meticulously probed *in vitro*. Finally, the *in vivo* therapeutic effect of the drug delivery system on COL-resistant *Salmonella* infections was studied using a mouse intraperitoneal infection model.

## 2 Materials and methods

### 2.1 Materials

Colistin (88%) was purchased from Hebei Shengxue Dacheng bio-pharmaceutical Co., Ltd. (Hebei, China), niclosamide (98%), cremophor EL (EL), ethyl oleate (EO), isopropyl myristate (IPM), Tween-80, Tween-20, and Poloxamer 188 were commercially available from Shanghai Macklin Biochemical Co., Ltd. (Shanghai, China). Poloxamer 407 (P407) was purchased from Accela ChemBio Co. Ltd. (Shanghai, China). Castor oil, octyl phenol, and polyoxyethylene ether-10 (OP) were purchased from Shanghai Yuanye Biochemical Co., Ltd. (Shanghai, China). Cremophor RH-40 was provided by Jiangsu Haian Petrochemical Plant (Jiangsu, China). Isopropanol, ethanol, propylene glycol, and polyethylene glycol 400 (PEG400) were obtained from Sinopharm Chemical Reagent Co., Ltd. (Shanghai, China). All other chemicals and reagents were of analytical grade. Ten non-duplicate clinical COL-resistant *Salmonella* strains were isolated from swine, chickens, and humans, specifically and preserved in the Laboratory of Pharmacology, Henan Agricultural University.

### 2.2 Antibacterial activity of the drug combination characterized using the checkerboard assay

The checkerboard assays were conducted to explore the synergistic activity between COL and NIC by determining the fractional inhibitory concentration index (FICI) (14). Briefly, COL was continuously diluted 2-fold along row by row, while NIC was continuously diluted 2-fold along the column by column to establish a matrix in 96-well plates, where each well contained different concentrations of two drugs. The bacterial suspension (100 μL) was added to each well to reach an ultimate bacterial concentration of approximately 5 × 10^5^ colony-forming unit (CFU)/mL. The 96-well plates were then incubated at 37°C for 18 h and examined for visible turbidity.

The FICI was calculated according to the following formula:

FICI=FICI_A_ + FICI_B_ = (MIC_A_ in combination/MIC_A_ alone) + (MIC_B_ in combination/MIC_B_ alone).

FICI was interpreted as follows: FICI ≤0.5 (synergy), 0.5 < FICI ≤1 (additive effect), 1 < FICI≤2 (irrelevance), FICI >2 (antagonism) (28).

### 2.3 Screening of nanoemulsion components by solubility assessment

The solubility of NIC in a range of oils (EO, IPM, and castor oil), surfactants (RH-40, EL, OP, Tween-80, and Tween-20), and co-surfactants (ethanol, isopropanol, propylene glycol, and PEG400) were screened for the development of nanoemulsion. An excess amount of NIC was added to a clean glass vial with 1 mL of each oil, surfactant, and co-surfactant and vortexed for 30 s (25). Then, the sample was mechanically shaken in a water bath shaker at 37°C for 72 h to reach equilibrium (29). The equilibrated samples were centrifuged at 10000 rpm for 15 min. The amount of soluble NIC of each sample was determined using an HPLC (high-performance liquid chromatography) method and UV–vis spectroscopy as described below. The oils, surfactants, and co-surfactants with the highest NIC solubility were selected as the components of nanoemulsions. All excipients used in this study are injectable and comply with an FDA approval.

### 2.4 Construction of pseudo-ternary phase diagram

Nanoemulsion was prepared by low energy (spontaneous) emulsification method. Based on the solubility results above, the selection was made for oil, surfactants, and co-surfactants to construct a pseudo-ternary phase diagram using the water titration method at room temperature. The specific experimental steps are as follows: first, the effect of surfactants on the formation of nanoemulsion was observed. Co-surfactant (ethyl alcohol) is separately mixed with surfactant (EL, Tween-20, and OP) to form a surfactant/co-surfactant mixture (Smix), and the Smix ratio is 3:1. Pseudo-ternary phase diagram was constructed with different ratios of oil to Smix (1:9, 2:8, 3:7, 4:6, 5:5, 6:4, 7:3, 8:2, 9:1) with EO as an oil phase. Similarly, the effects of different co-surfactants on the formation of nanoemulsion were analyzed. Then, EL was used as the surfactant, and it was mixed with ethyl alcohol at different Smix (1:1, 2:1, 3:1, and 3:2) with EO as the oil phase to determine the maximum microemulsion region from four different ratios of Smix. Then, these samples were checked for transparency and uniformity after the gradual addition of water (30).

### 2.5 Preparation and optimization of COL/NIC-NEs

**Figure 1:**
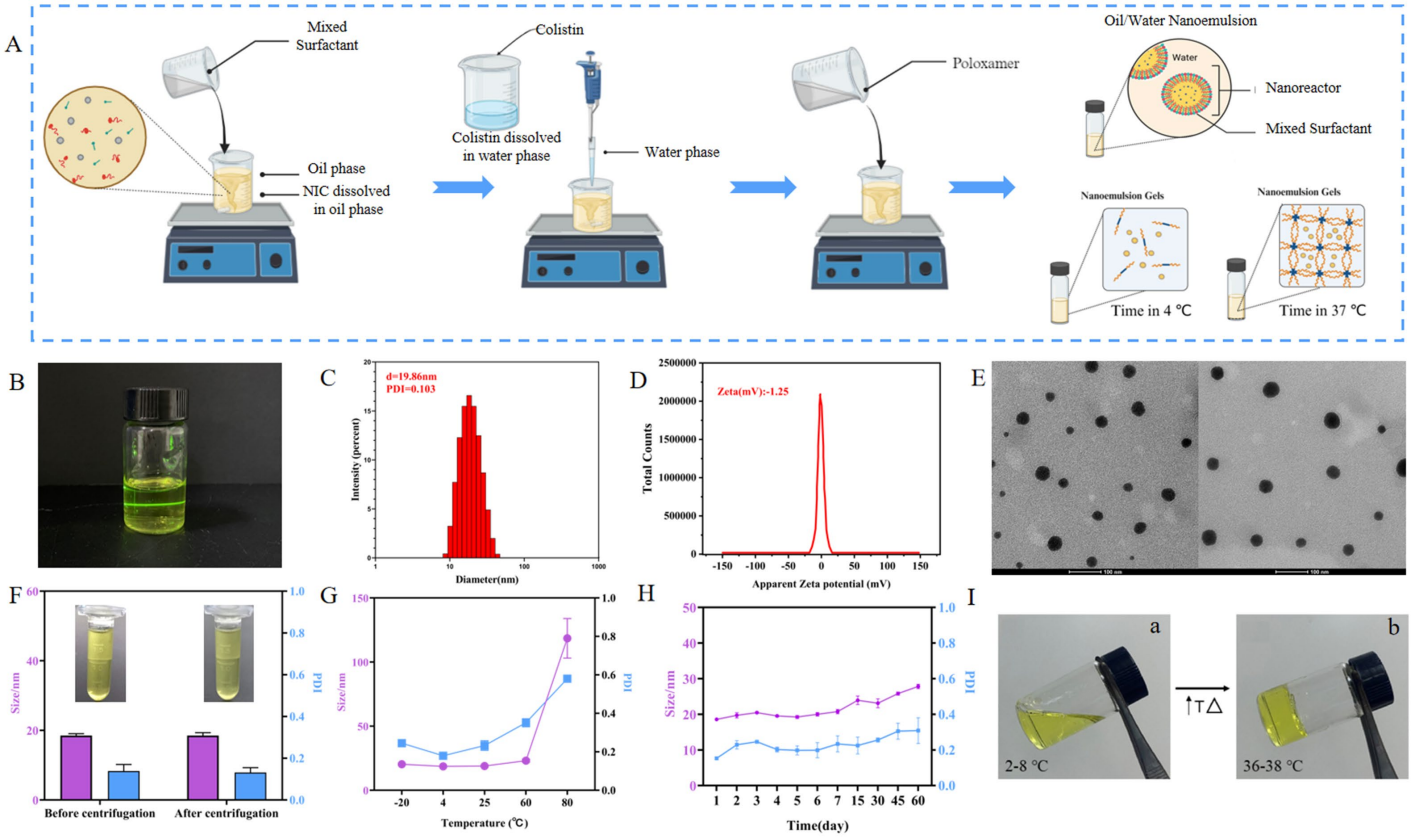
Characterization of COL/NIC-NEs. **(A)** Schematic presentation of the process of preparation of the COL/NIC-NEs and COL/NIC-NEGs. **(B)** Schematic diagram of the structure of COL/NIC-NEs. **(C)** Particle diameter and PDI of COL/NIC-NEs. **(D)** Zeta of COL/NIC-NEs. **(E)** TEM diagram of COL/NIC-NEs. **(F)** Centrifugation stability of COL/NIC-NEs. **(G)** Cold–hot alternate stability of COL/NIC-NEs. **(H)** Storage stability of COL/NIC-NEs. **(I)** Representative pictures of COL/NIC-NEGs at 2–8°C (storage temperature) (a) and 36–38°C (body surface temperature) (b).

The microemulsion technique was optimized to produce blank nanoemulsions by adjusting the ratio of EO (oil phase), EL (surfactant), and ethyl alcohol (co-surfactant). The optimization process was guided by particle size and polydispersity index (PDI) as key indicators. Six different blank nanoemulsions (NE1 to NE6) were prepared (Supplementary Table S1). Particle size and PDI were measured by zeta-sizer Nano (ZS-90, Malvern Instruments, Malvern, UK). Using the optimized conditions of NE6, COL/NIC-NEs were prepared based on blank nanoemulsion formulations. Briefly, NIC (0.2% w/w) dissolved in EO (5%) was added to Smix (45% w/w) consisting of EL/ethyl alcohol (3:1) and stirred using a magnetic stirrer for 5 min. Finally, 5 mL of deionized water containing (0.4%) COL was added slowly with stirring to obtain a clear solution. A schematic diagram for the fabrication of COL/NIC-NEs and COL/NIC-NEGs is shown in Figure 1A.

### 2.6 Characterization of the COL/NIC-NEs

#### 2.6.1 Droplet size, polydispersity index (PDI), and zeta potential analysis

The particle size, polydispersity index (PDI), and zeta potential were measured using Zeta-sizer Nano ZS-90 (Malvern Instruments, MAL, United Kingdom). Each measurement (100 runs) was performed in triplicate at 25°C and an equilibration time of 60 s.

#### 2.6.2 Morphology observation

The morphological characteristics and structure of the provided COL/NIC-NEs were observed using TEM (transmission electron microscope) (31). COL/NIC-NEs were diluted with deionized water (1:100). Appropriate samples were placed on a carbon-coated copper grid and dried at 25°C for 10 min, then negatively stained with 2% (w/v) phosphotungstic acid solution, and finally observed under TEM at 120 kV (TEM, Czech, Tecnai G2 Spirit Bio).

#### 2.6.3 Stability study

For the research of new drug dosage forms, the stability of nanoemulsion would be one of the most important properties. Here, the stability of the COL/NIC-NEs was evaluated by centrifugal tests, temperature factors, and preliminary stability for 3-month storage. At the same time, the particle size and PDI of the COL/NIC-NEs were measured before and after centrifugation.

### 2.7 Incorporation of nanoemulsion into gels

The incorporation of nanoemulsion into a mixture of 30% P407 and 8% P188 was carried out by first preparing the poloxamer gel base. To do this, 30% P407 and 8% P188 powder were added to distilled water while stirring at a low speed. The mixture was then allowed to hydrate for 10 min before increasing the stirring speed too high for an additional 30 min to ensure proper gel formation. The prepared nanoemulsion was then added to the poloxamer gel at a concentration of 50% (v/v) and mixed until a homogenous gel was formed. We then allowed the mixture to sit for 24 h to allow for proper hydration and formation of the gel. Finally, we checked the gelation temperature and pH of the final product to ensure it met the desired specifications.

### 2.8 Fourier transform infrared (FT-IR) radiation measurements

Fourier transform infrared spectrometer (Thermo Scientific Nicolet, iS20 FTIR, USA) was performed to check the chemical composition of the formulation. The specific parameters are based on previous studies (25). The measurement range is 350 to 4,000 wave numbers, the data interval is 0.5 cm^−1^, and the resolution is 2 cm^−1^. The figure is expressed as a relation between relative transmittance (%) and wave number (cm^−1^).

### 2.9 Determination of niclosamide and colistin

A high-performance liquid chromatography system (Agilent Technology Co., Ltd) was used to detect the content of NIC according to the previously described method (32). NIC was separated by an Agilent TC-C18 column (5 mm, 4.6 mm–250 mm) guarded with a precolumn at 30°C and detected at 330 nm. The injection volume was set to 20 μL. The mobile phase consisted of 85% methanol and 15% water (containing 0.1% formic acid) pumped at a flow rate of 1.0 mL/min. An NIC stock solution was prepared in methanol (1 mg/mL) and sequentially diluted with methanol to 100, 80, 60, 40, and 20 μg/mL. A standard curve was established, and the content was calculated using the peak area of the sample at the retention time (6.8 min).

COL content was determined using UV–vis spectroscopy. COL solution (1 mg/mL in acetonitrile) was diluted with distilled water to 500, 250, 125, 62.5, 1, 0.5, and 0.25 μg/mL, and the absorption of the sample at 230 nm was determined using a UV–vis spectrometer. A standard curve was established, and the content was calculated using the absorption value of the sample.

### 2.10 *In vitro* drug release study

*In vitro* release studies of COL and NIC from COL/NIC-NEs and COL/NIC-NEGs were carried out at 37°C via dialysis as previously described (33). The COL/NIC-NEs and COL/NIC-NEGs (1 mL) were transferred to dialysis bags (10 kDa MWCO), respectively, and then immersed in 20 mL of PBS (pH 7.4) and kept in a shaking water bath at 37°C and 100 rpm. At a predetermined time (0.5, 1, 1.5, 2, 4, 6, 8, 10, 12, 14, 24, 36, 48, 60, 72, 84, and 96 h), the 0.5 mL COL/NIC-NEs and COL/NIC-NEGs in the dialysis bag was taken out and refilled with PBS of equal volume after each sampling. The accumulative release of NIC and COL from COL/NIC-NEs and COL/NIC-NEGs was determined by HPLC and UV–vis spectroscopy. All experiments were performed in triplicate.

### 2.11 Minimal inhibitory concentration (MIC) assay

The MICs of the COL/NIC-NEs and COL/NIC-NEGs to the 10 COL-R strains were measured by broth microdilution method according to the Clinical and Laboratory Standards Institute guidelines (34), and *E. coli* ATCC 25922 was used as the quality control. All drugs were diluted using 2-fold serial dilutions in a sterile 96-well microtiter plate with Mueller-Hinton broth (MHB), and then, 100 μL of the bacterial suspensions (1 × 10^6^ CFUs/mL) was added to each well. MIC values were defined as the lowest concentrations of drugs with no visible growth of bacteria after 18 h of incubation at 37°C.

### 2.12 Time-kill curves

To further investigate the bactericidal activity of the COL/NIC-NEs and COL/NIC-NEGs, the time–kill curves of randomly selected clinical COL-resistant *Salmonella* SH134 (*mcr-1* positive) isolate and S2a (*mcr-1* negative) were determined during the exponential growth phase as described with some modifications (14). Bacterial cells at a final concentration of 1 × 10^5^ CFUs/mL were cultured with MHB in NIC (1 μg/mL), COL (2 μg/mL), blank-NEs, and the COL/NIC-NEs and COL/NIC-NEGs (containing COL and NIC at 2 and 1 μg/mL, respectively). Samples of the bacterial cells were collected after culturing for 0, 2, 4, 8, 12, and 24 h and spread on LB agar plates after dilution. CFUs were calculated after incubation at 37°C overnight. All experiments were repeated three times on different days.

### 2.13 Fluorescence assay

To investigate the antibacterial mechanisms of COL/NIC-NEs and COL/NIC NEGs, we conducted fluorescence experiments. Bacterial pretreatments in all measurements were performed with similar protocols as follows. In the fluorescence assay, the *mcr-1*-positive strain SH134 was chosen as the indicator strain. Briefly, bacteria were grown overnight at 37°C with shaking at 200 rpm. Then, the cultures were washed and suspended with 5 mmol/L HEPES (pH 7.0, plus 5 mmol/L glucose). The OD_600_ of bacteria suspension was standardized to 0.5 in the same buffer, and fluorescent dye was added. After incubation at 37°C for 30 min, an aliquot of 1 mL of bacterial suspension was mixed with the same concentration of free COL, free NIC, free COL and NIC, blank-NEs, COL/NIC-NEs, and COL/NIC-NEGs. After incubation for 1 h, 200 μL of bacterial cells was added to the 96-well plate. Subsequently, fluorescence intensity was measured on a Spark 10 M Microplate reader (Tecan) (35).

#### 2.13.1 Outer membrane permeability assay

Fluorescent probe 1-Nphenylnaphthylamine (NPN) was used to evaluate the permeability of the bacterial outer membrane (OM). Fluorescence intensity was measured with the excitation wavelength at 350 nm and emission wavelength at 420 nm (36).

#### 2.13.2 Cell membrane integrity assay

The fluorescence intensity of propidium iodide (PI)-labeled (Beyotime, Shanghai, China) in the presence of increasing drugs was measured with an excitation wavelength of 535 nm and emission wavelength of 615 nm.

#### 2.13.3 Proton motive force assay

PMF consists of two components, including the membrane potential (Δψ) and the transmembrane proton gradient (ΔpH). The membrane potential of *Salmonella* SH134 was measured with 3,3-dipropylthiothiobromocyanide (DiSC3 (5), 0.5 μM) (Aladdin, Shanghai, China). Fluorescence intensity was measured at an excitation wavelength of 622 nm and an emission wavelength of 670 nm. The pH gradient (ΔpH) was determined by applying the pH-sensitive fluorescent probe BCECF-AM (20 μM) (Beyotime, Shanghai, China). The excitation and emission wavelengths on the fluorescence spectrometer were set to 500 and 522 nm, respectively (37).

#### 2.13.4 Efflux pump assay

The fluorescence dye ethidium bromide (EtBr 5 μM) was used to assess the inhibitory effect of COL/NIC-NEs and COL/NIC-NEGs on the activities of efflux pumps. The well-known efflux pump inhibitor CCCP (100 μM) was used as a positive control. The efflux of EtBr from bacterial cells was measured using an excitation wavelength of 530 nm and an emission wavelength of 600 nm.

#### 2.13.5 Total ROS measurement

2′,7′-Dichlorodihydrofluorescein diacetate (DCFH-DA, 10 μM) was used to monitor the levels of ROS in *Salmonella* strain (13). Briefly, the bacteria suspension was exposed to various groups of treatment for an additional 30 min, and the supernatant of the mixture was mixed with chromogenic solution from a ROS Assay Kit (Beyotime, China) according to the manufacturer instructions. Fluorescence intensity was monitored after 30 min of incubation at excitation/emission of 488/525 nm at 37°C.

### 2.14 *In vivo* antibacterial activity against colistin-resistant infection

Fifty-six KM mice were randomly divided into seven groups (*n* = 8) for survival experiments. All mice were treated with streptomycin by gastric administration 3 days before the formal experiment. Each group was infected with *Salmonella* SH134 at 1.0 × 10^7^ CFUs by intraperitoneal injection administration in addition to the blank control group. Each group was intraperitoneally injected once a day for 3 days with the following methods: PBS, model, free COL (5 mg/kg), free NIC (5 mg/kg), free COL+NIC (COL 5 mg/kg, NIC 2.5 mg/kg), and COL/NIC-NEs (COL 5 mg/kg, NIC 2.5 mg/kg). Particularly worth mentioning is that the COL/NIC-NEGs (COL 10 mg/kg, NIC 5 mg/kg) group was treated by injection only on the first day. The survival rate of mice in each group was observed for up to 5 days.

Another six groups (*n* = 6) with a sublethal dose of *Salmonella* SH134 at 1.0 × 10^6^ CFUs received the same treatment as described above. Colony colonization and histopathology of the liver and spleen were studied. All mice were killed by cervical dislocation on the second day. The tissue samples were ground for continuous dilution and plated on SS agar plates to observe the number of colonies. The typical lesions of livers or spleens of different groups of mice were prepared by hematoxylin–eosin (HE) staining, and histopathological analysis was performed.

### 2.15 Statistical analysis

All experiments were performed in triplicate. Statistical analysis was performed using GraphPad Prism 8.0. All values are stated as mean ± SD. Unpaired *t*-tests (normally distributed data) between two groups and one-way ANOVA among multiple groups were used to calculate *p*-values. Statistical significance was determined at *<*p* 0.05, **p<0.01, and ***p<0.001; ns denotes no significant difference.

## 3 Results

### 3.1 Synergistic antibacterial effects of niclosamide on colistin-resistant *Salmonella*

**Figure 2:**
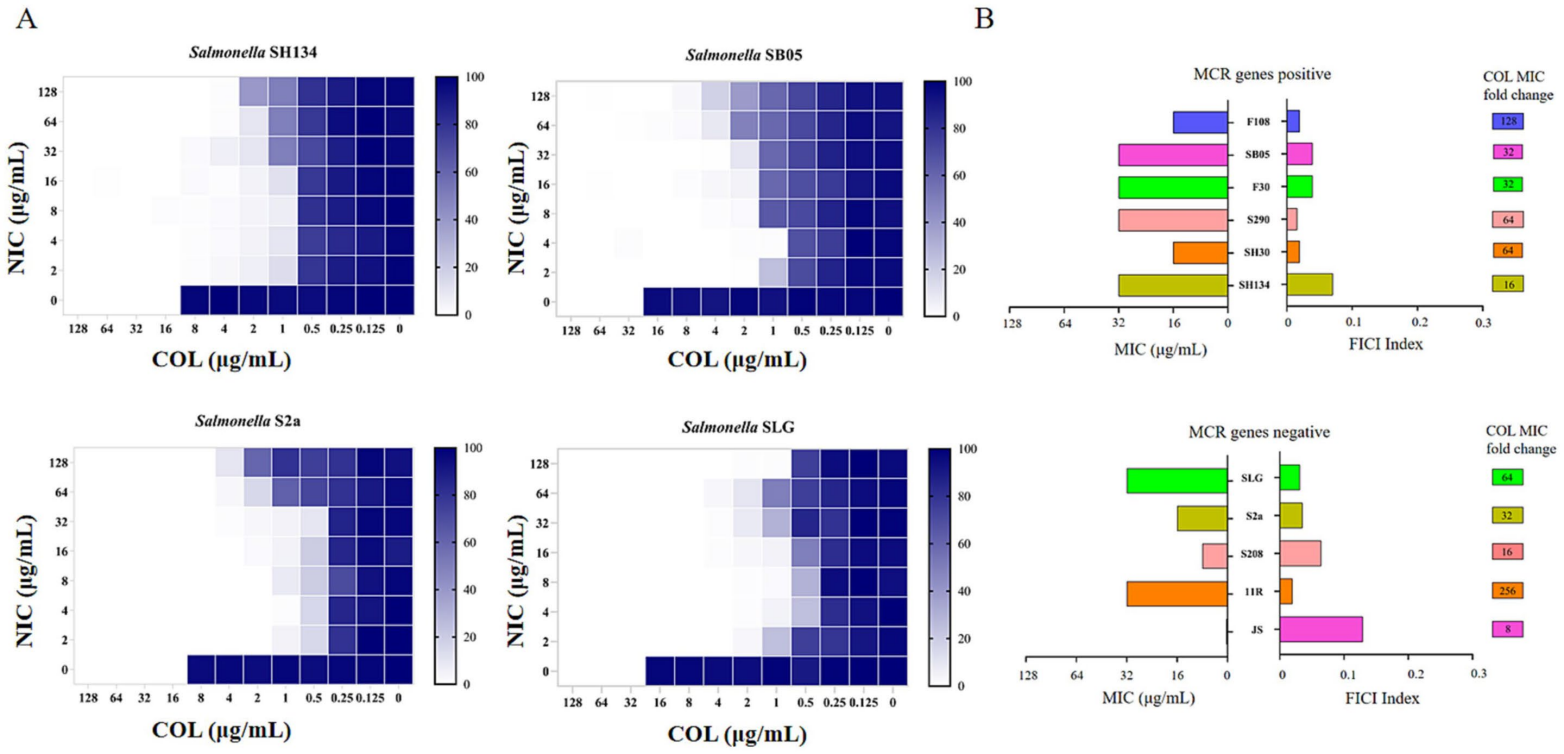
Niclosamide drastically potentiates colistin activity against colistin-resistant *Salmonella*. **(A)** Checkerboard assays between niclosamide and colistin against *Salmonella* SH134, *Salmonella* SB05, *Salmonella* S2a, and *Salmonella* SLG. Dark-blue regions represent higher cell density. 0 implied no bacteria growth and 100% implied that all bacteria survive. Data represent the mean absorbance of two biological replicates. **(B)** Antibacterial activity (MIC, μg/mL) and synergistic activity of niclosamide with colistin (FIC index) against diverse species of *mcr-1* gene positive and *mcr-1* genes negative.

Checkerboard assays were used to evaluate the antibacterial activity of a combination of NIC and COL against COL-resistant *Salmonella* (13). As shown in Figure 2A, a synergistic effect was observed in *mcr-1*-positive and *mcr-1*-negative strains. NIC robustly reversed COL resistance, with the MIC of COL decreased to a sensitivity of no more than 2 μg/mL, with FIC indices lower than 0.5 for all the tested resistant strains (FICI = 0.0195–0.129) (Figure 2B and Table 1). Importantly, the MIC of COL in combination ranging from 0.0625 to 1.0 μg/mL was lower than the resistance breakpoint, which was set by the Clinical and Laboratory Standards Institute at 4 to 8 μg/mL for Gram-negative bacteria. These results demonstrated that the NIC can reverse the resistance phenotype of COL-resistant *Salmonella*.

**Table 1:** Minimum inhibitory concentration (MIC) and fractional inhibitory concentration index (FICI) of colistin and niclosamide in mono- or combination treatment of colistin-resistant bacteria determined using the checkerboard assay.

Species	Strain	Source	mcr-1	MIC value (μg/mL)				
				Free colistin	Free niclosamide	Colistin in combination	Niclosamide in combination	FICI
*Salmonella*	SH134	Pig	+	16	512	1	4	0.0703
	SH30	Pig	+	16	512	0.25	2	0.0195
	S290	Pig	+	32	512	0.25	4	0.0156
	F30	Pig	+	32	512	1	8	0.0391
	SB05	Pig	+	32	512	1	4	0.0391
	F108	Chicken	+	16	512	0.125	8	0.0195
	S2a	Chicken	−	16	512	0.5	2	0.0351
	11R	Human	−	32	512	0.125	8	0.0195
	S208	Chicken	−	8	512	0.5	1	0.0644
	SLG	Chicken	−	32	512	0.5	8	0.0312
	JS	−	−	0.5	512	0.0625	2	0.129

### 3.2 Measurement of niclosamide solubility

**Figure 3:**
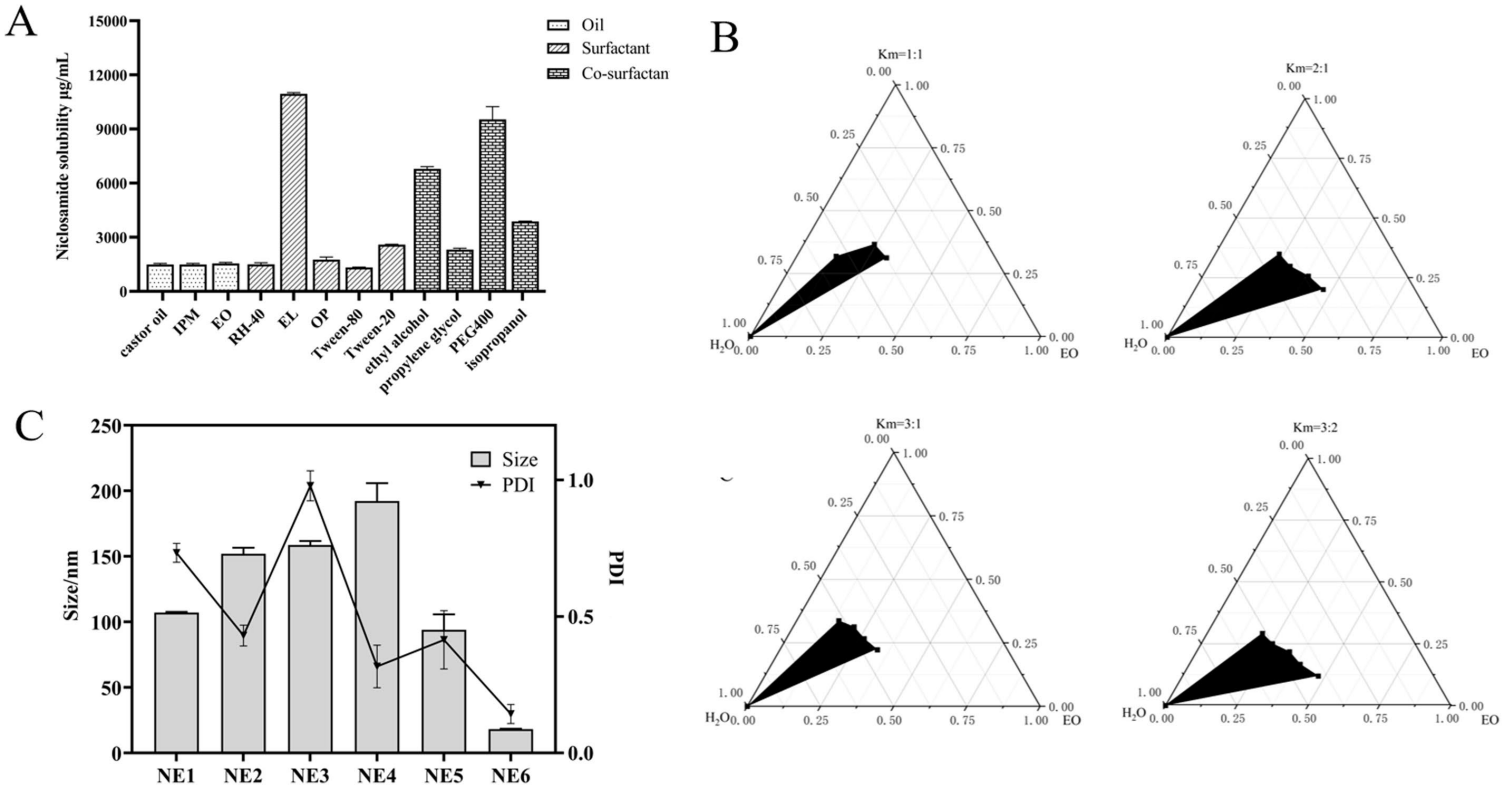
Screening and optimization of components in nanoemulsion. **(A)** Drug solubility determination by adding an excess amount of niclosamide per mL of various oils (EO, castor oil, and IPM), surfactants (RH-40, EL, OP, Tween-80, and Tween-20), and co-surfactants (ethyl alcohol, propylene glycol, PEG400, and isopropanol) in separate vials (*n* = 3 ± SD). **(B)** Pseudo-ternary phase diagrams of EO, EL, and ethyl alcohol indicate the o/w nanoemulsion region at Smix (1:1), Smix (2:1), Smix (3:1), and Smix (3:2). **(C)** Average particle size and PDI of different formulations.

The drug absorption and bioavailability of NIC when administered in combination with COL *in vivo* may be limited by its poor solubility. The selection of oil phase, surfactant, and co-surfactant with high solubility is the basis of the successful preparation of nanoemulsion. Oil phase drug solubilizing capacity is a crucial characteristic as it affects the potential of formulation for drug loading. As shown in Figure 3A, among three oil phases, the solubility of NIC in EO (1.54 ± 0.04 mg/mL) is a little more than that in IPM (1.49 ± 0.06 mg/mL) and castor oil (1.52 ± 0.06 mg/mL). Moreover, due to the advantages of strong permeability, easy absorption, high stability, good safety, and low toxicity of EO, EO was selected for further investigation for nanoemulsion studies as oil bases (38). Surfactant EL had the highest solubility (10.92 ± 0.64 mg/mL) of NIC among the different surfactants, followed by Tween-20 (2.63 ± 0.05 mg/mL), OP (1.77 ± 0.1 mg/mL), and Tween-80 (1.35 ± 0.05 mg/mL). Among the different co-surfactants, PEG400 and ethyl alcohol showed higher solubility than the isopropanol and propylene glycol. Thus, we selected oil phase (EO), surfactant (EL, Tween-20, and OP), and co-surfactant (PEG400, ethyl alcohol, and isopropanol) for subsequent pseudo-ternary phase diagram screening.

### 3.3 Construction of phase solubility diagrams, selection of nanoemulsion components, and preparation of COL/NIC-NEs

The pseudo-ternary phase diagram, which compares the microemulsion region, is an effective evaluation technique (39). As shown in Supplementary Figure S1, surfactant EL had the largest microemulsion area compared with Tween-20 and OP, indicating that EL is a surfactant suitable for NIC nanoemulsions. According to Supplementary Figure S2, a comparison of the microemulsion region of ethyl alcohol, PEG400, and isopropanol, with the biggest microemulsion region, was chosen as the co-surfactant for NIC nanoemulsions. Surfactant/co-surfactant (Smix) ratio plays a very important role in preparing more stable and effective nanoemulsions. As shown in Figure 3B, pseudo-ternary phase diagrams were constructed by using different Smix ratios (1:1, 2:1, 3:1, and 3:2). The results show that the microemulsion region is the largest when the Smix ratio is 3:1, which indicates that the formation of nanoemulsion is the strongest, so it can be used in the final formulation. Based on the pseudo-ternary phase diagrams, EO, EL, and ethyl alcohol were chosen, respectively, as oil phase, surfactant, and co-surfactant of NIC nanoemulsions.

Blank nanoemulsions were optimized based on different proportion factors in the prescription. Significant changes were observed by changing these prescriptions in different proportions. The final formulation was prepared based on the optimized blank nanoemulsion formula (NE1 to NE6). As shown in Figure 3C and Supplementary Table S2, NE6 has the characteristics of small particle size and small PDI, compared with other formulations, so NE6 is selected as the final formulation of the COL/NIC-NEs.

### 3.4 Characterization of the COL/NIC-NEs

The COL/NIC-NEs were prepared according to the optimized formulation NE6. The preparation process of COL/NIC-NEs and COL/NIC-NEGs is illustrated in Figure 1A. The appearance of the COL/NIC-NEs as shown in Figure 1B was yellow, clear, and transparent liquid. The mean particle sizes, PDI, and zeta potentials of the COL/NIC-NEs were measured using DLS. The average hydrodynamic diameter of the COL/NIC-NEs droplets was about 19.86 ± 2.21 nm, with a PDI of 0.103 ± 0.01 (Figure 1C), and the zeta potential of the COL/NIC-NEs was −1.25 mv (Figure 1D), indicating that the nanoemulsions had satisfactory quality results. From the images obtained by TEM analysis, it is evident that COL/NIC-NEs showed spherical, non-adhesive, non-aggregating, and evenly distributed in space, the sizes of the nanodroplets corresponded to the ones measured with DLS, as evident from Figure 1E.

To investigate the stability of the COL/NIC-NEs, we conducted centrifugation stability, temperature stability, and long-term storage stability tests. The centrifugal stability study found that after centrifugation, the COL/NIC-NEs did not layer or precipitate, and remained a yellow clear transparent liquid with no significant changes in particle size and PDI, indicating that the formula has good centrifugal stability (Figure 1F). In the study of temperature stability (Figure 1G), the COL/NIC-NEs remained stable at low temperatures of −20°C, refrigerated temperature of 4°C, room temperature of 25°C, and 60°C, exhibiting minimal changes in particle size and PDI. However, after treatment at 80°C, the particle size of COL/NIC-NEs significantly increased from 19.34 nm to 112.6 nm, indicating that the COL/NIC-NEs are unstable at 80°C and high temperatures can disrupt the dispersion system of the nanoemulsion. Long-term storage stability is a necessary precondition for the practical application of nanoemulsions. As shown in Figure 1H, the long-term stability experiment found that the COL/NIC-NEs system remained relatively stable within 1–7 days, with a particle size of around 20 nm. Over longer storage periods, it was found that the particle size slowly increased. However, despite the rise, the sizes of COL/NIC-NEs were still in the acceptable range of nanoemulsion.

### 3.5 Fabrication and validation of COL/NIC-NEs loaded gel

Following the optimization studies, a thermal sensitivity situ gel loaded with COL/NIC-NEs was prepared. Then, the evaluation of the COL/NIC-NEGs of poloxamer 407 and 188 for temperature was carried out at two different temperatures, i.e., 2–8°C and 36–38°C, respectively, to verify the thermosensitivity of the *in situ* gels. As shown in Figure 1I, the COL/NIC-NEGs remained at a transparent solution state at 2–8°C and the solution state of the COL/NIC-NEGs transformed into the gel state at 36–38°C. The pH of the COL/NIC-NEGs measured by the PH meter is 6.51, which meets the requirements of injection preparations. The results show that the prescription conditions of the COL/NIC-NEGs are reasonable and feasible.

### 3.6 FTIR

**Figure 4:**
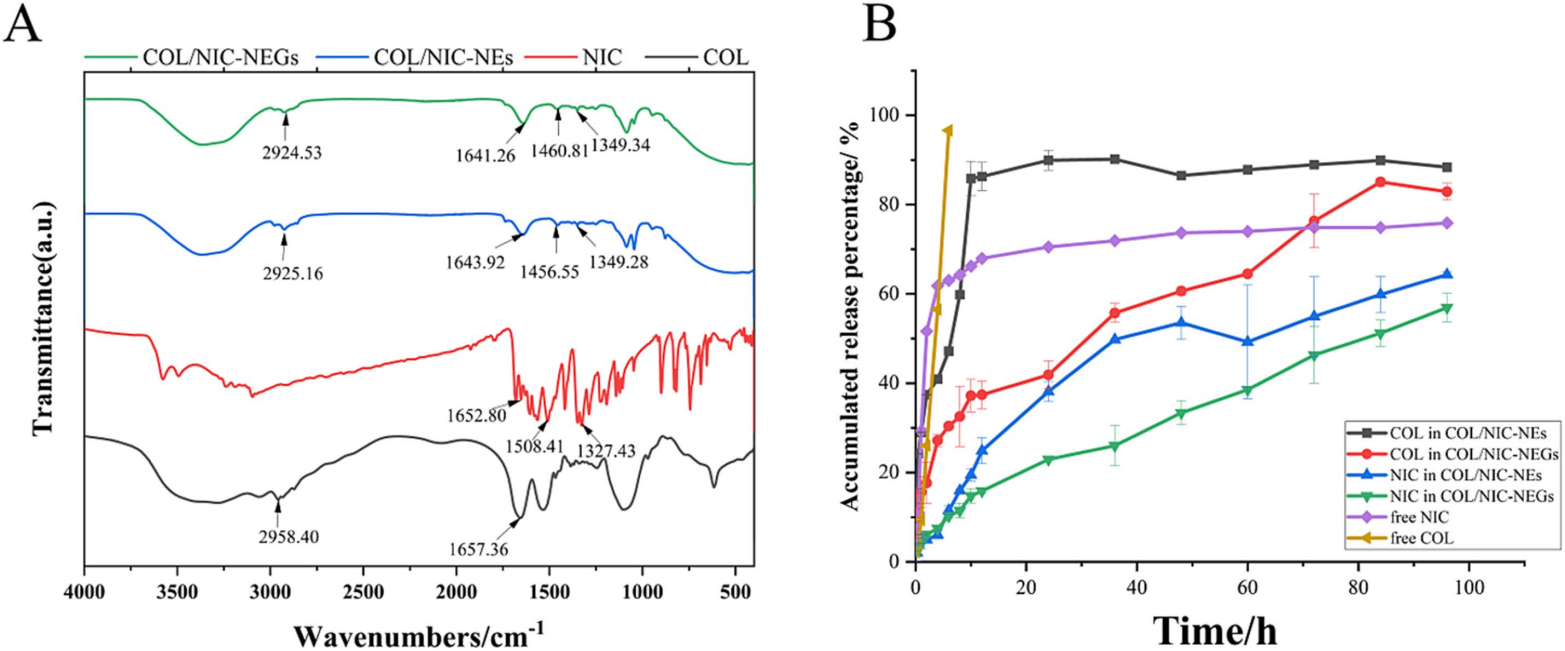
**(A)** FTIR spectra of niclosamide (red), colistin (black), COL/NIC-NEs (blue), and COL/NIC-NEGs (green). **(B)** Cumulative *in vitro* drug released study of free colistin and niclosamide and COL/NIC-NEs and COL/NIC-NEGs in pH 6.8 PBS at 37°C (*n* = 3 ± SD). The COL/NIC-NEs and COL/NIC-NEGs showed sustained drug release over 96 h relative to the control solution.

As shown in Figure 4A, the COL spectrum (black) showed characteristic peaks at 1657 cm^−1^ (−C=O amide bonds) and 2,958 cm^−1^ (−CH stretching). The characteristic FTIR spectrum of NIC (red) signaling includes bending vibration of phenolic hydroxyl groups (−C − O), −NO2 bending, and − C=O stretching bending, which occur at 1327–1329, 1,508–1,510, and 1,652–1,653 cm^−1^, respectively. Major peaks of the COL/NIC-NEs and COL/NIC-NEGs appeared at wave numbers 2,924–2,925, 1,641–1,643, 1,456–1,460, and 1,349 cm^−1^. The FTIR spectrum of COL/NIC-NEs (blue) and COL/NIC-NEGs (green) showed lower peak intensities, which suggests a good blend of COL and NIC with the selected excipients without the presence of chemical interactions.

### 3.7 *In vitro* drug release studies

To investigate the extended-release effect, we evaluated the NIC and COL-release from the COL/NIC-NEs and COL/NIC-NEGs at 37°C in PBS (pH 7.4) (Figure 4B). The release profile demonstrated an initial burst release of free COL and NIC within the 6 h (96.63 ± 2.31% and 61.82 ± 1.85%, respectively). The release rate of COL in COL/NIC-NEs and COL/NIC-NEGs was 88.35 ± 0.38% and 82.88 ± 1.87%, respectively, at 96 h. Similarly, the release rate of free NIC was 75.89 ± 0.56%, that of COL/NIC-NEs was 64.31 ± 0.52%, and that of COL/NIC-NEGs was 56.92 ± 3.19%. After fitting, COL and NIC in COL/NIC-NEs conform to the first-order release model (adjusted R^2^ = 0.91923 and 0.98597, respectively), and the equation that COL and NIC in COL/NIC-NEGs conform to is the Higuchi model (adjusted R^2^ = 0.98644 and 0.97862, respectively) (Supplementary Figure S3). As expected, the release of drugs NIC and COL from COL/NIC-NEs and COL/NIC-NEGs was slower than the free NIC and COL. It shows that NIC- and COL-loaded nanoemulsions and nanoemulsion gels have slow-release effects, achieving the effect of reducing the amount and increasing the efficiency.

### 3.8 Antibacterial activity of the COL/NIC-NEs and COL/NIC-NEGs *in vitro*

**Figure 5:**
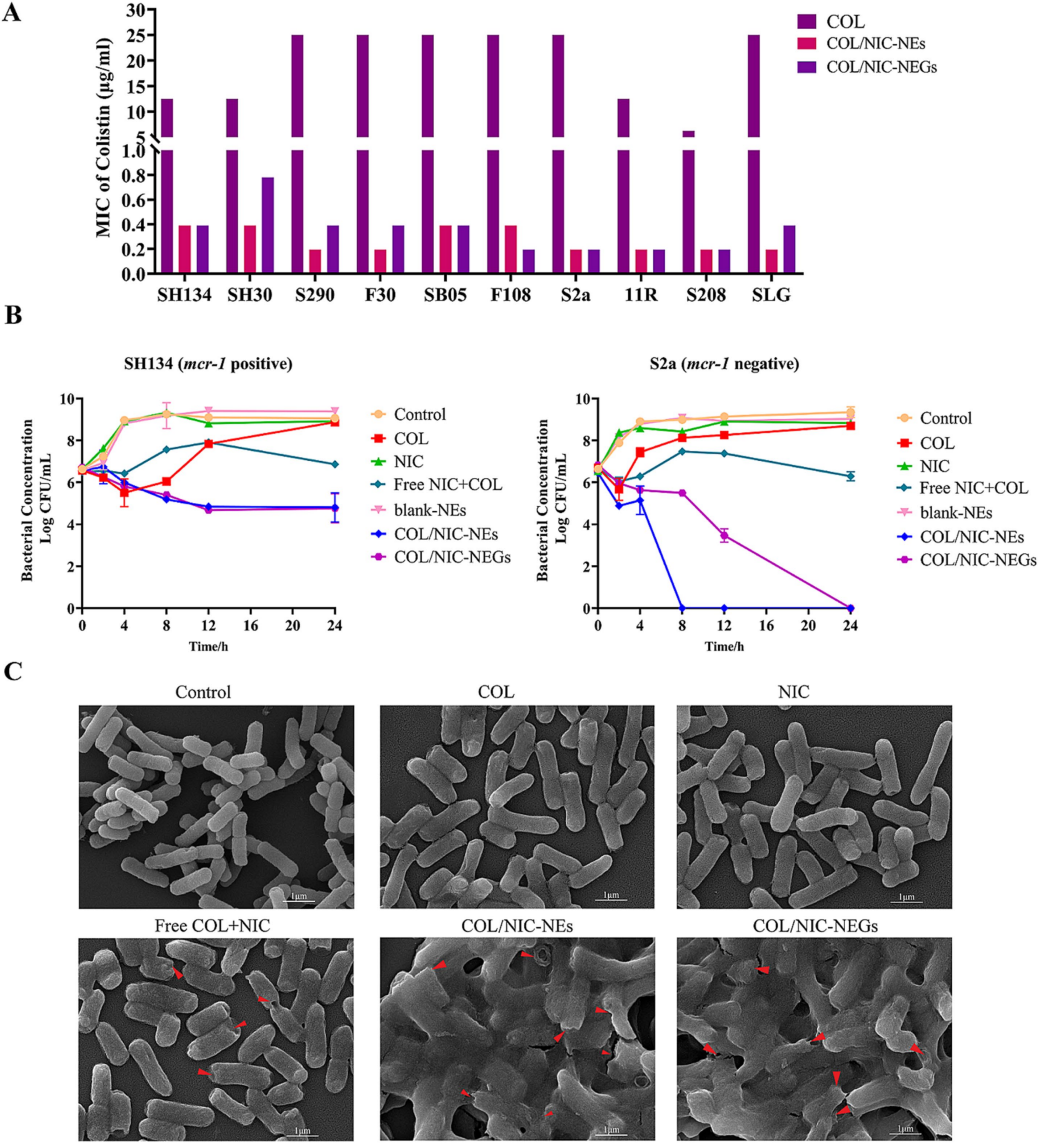
Antibacterial activity of COL/NIC-NEs and COL/NIC-NEGs. **(A)** MIC values of colistin, colistin in COL/NIC-NEs and COL/NIC-NEGs against different bacterial strains. The bar graphs show the MIC values of colistin for the tested bacterial strains. The initial concentration of colistin and colistin in COL/NIC-NEs and COL/NIC-NEGs is 2000 μg/mL, respectively. **(B)** Killing kinetics of colistin-resistant *Salmonella* treated with various formulations (free COL, 2 μg/mL; free NIC, 1 μg/mL; free COL+NIC: COL 2 μg/mL, NIC 1 μg/mL; blank-NEs; COL/NIC-NEs and COL/NIC-NEGs containing COL at 2 μg/mL and NIC at 1 μg/mL). **(C)** Scanning electron microscopic (SEM) images illustrating the morphological change of the bacterial cell membrane of *Salmonella* SH134 after various treatments.

To study the antibacterial effects of COL/NIC-NEs and COL/NIC-NEGs, we examined the MIC of the COL, NIC, COL/NIC-NEs, and COL/NIC-NEGs, respectively (Supplementary Table S2). Compared with alone COL, the MIC of COL in the combination of the COL/NIC-NEs and COL/NIC-NEGs was decreased significantly, which was reduced by 16-128-fold (Figure 5A). Excitingly, a synergistic effect in the COL/NIC-NEs and COL/NIC-NEGs was observed in all the tested *mcr-1*-positive and *mcr-1-*negative COL-resistant strains. To further evaluate the synergetic effect of the COL/NIC-NEs and COL/NIC-NEGs, we performed the time–kill curves assays using *mcr-1*-positive strain SH134 and *mcr-1*-negative strain S2a as a pattern strain. The results suggested that the COL/NIC-NEs and COL/NIC-NEGs exhibited synergistic bactericidal efficacy. Specifically, neither alone NIC nor COL killed *Salmonella* SH134 and *Salmonella* S2a in the medium. However, the COL/NIC-NEs and COL/NIC-NEGs decreased CFUs of *Salmonella* SH134 and *Salmonella* S2a approximately by >2-log_10_ in 24 h compared to using any drug alone (Figure 5B). Furthermore, the COL/NIC-NEs and COL/NIC-NEGs can enhance the bactericidal activity of COL compared with free COL+NIC (Figure 5B; Supplementary Figure S4). The enhanced antibacterial activity of the COL/NIC-NEs and COL/NIC-NEGs was further confirmed by examining the bacterial surface morphology using scanning electron microscopy (SEM) (Figure 5C). SEM images showed rough bacterial surfaces or lytic bacterial cells destroyed and damaged by the COL/NIC-NEs and COL/NIC-NEGs treatment (arrows in Figure 5C). In contrast, the cell membrane of bacterial cells remained relatively intact after treatment with the free drug combination or alone. In conclusion, both the COL/NIC-NEs and COL/NIC-NEGs significantly enhance the antibacterial activity of the drug combination.

### 3.9 Antibacterial mechanism of COL/NIC-NEs and COL/NIC-NEGs

**Figure 6:**
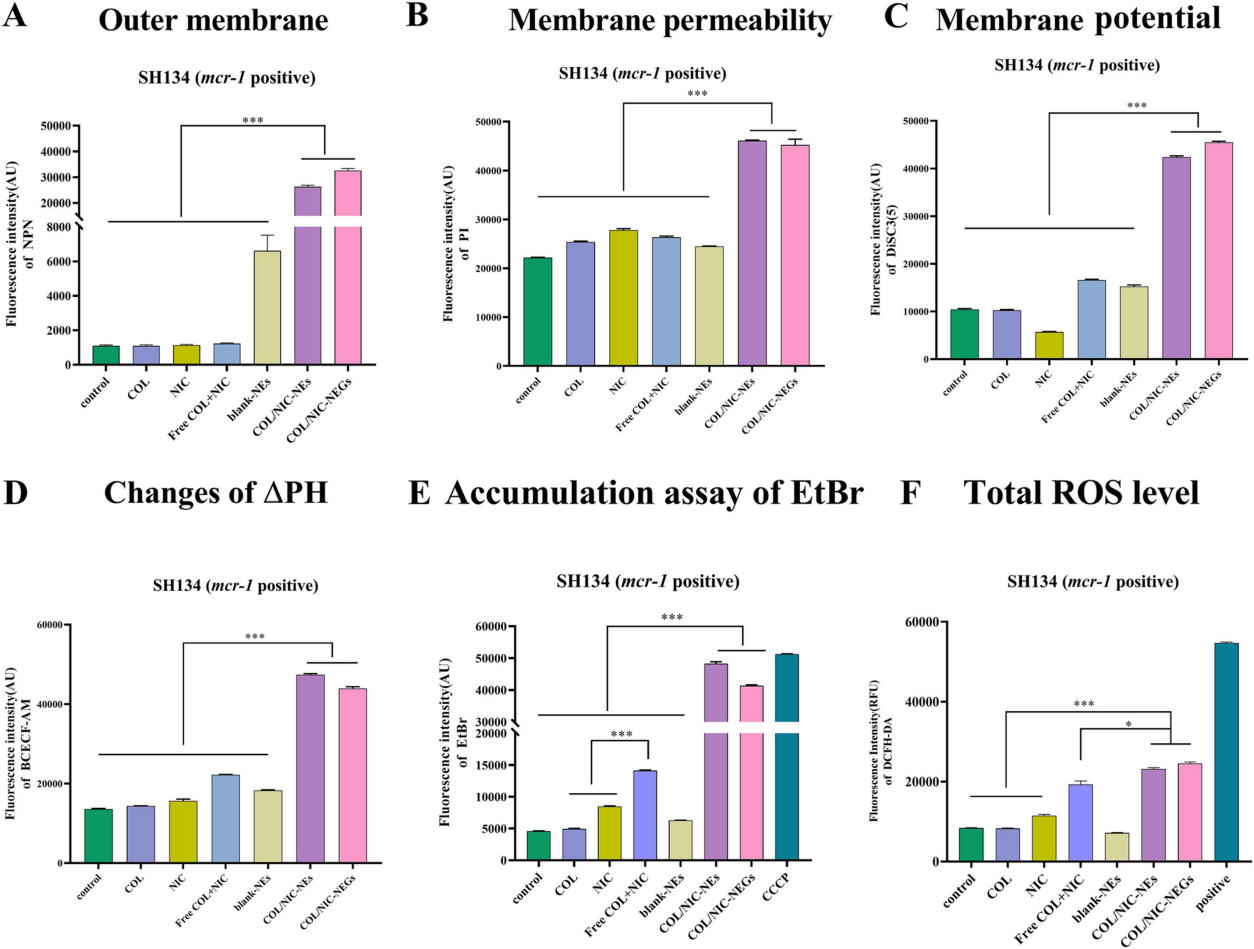
Antibacterial mechanisms of COL/NIC-NEs and COL/NIC-NEGs against colistin-resistant *Salmonella*. **(A)** Effects of different treatments on the permeability of bacterial outer membrane investigated using 1-N-phenylnaphthylamine (NPN; *n* = 3). **(B)** Effects of different treatments on the permeability of bacterial membrane permeability investigated using PI (*n* = 3). Effects of different treatments on the PMF by measuring the membrane potential (Δψ) **(C)** and pH gradient (ΔpH) **(D)**. **(E)** Ethidium bromide (EtBr) accumulation in untreated SH134 (control), COL, NIC, free COL+NIC, blank-NEs, COL/NIC-NEs and COL/NIC-NEGs, and 10 μm CCCP (positive control) reflect the efflux pump effect. **(F)** Excessive ROS produced in *Salmonella* SH134 after COL/NIC-NEs and COL/NIC-NEGs. All data are presented as mean ± SD, and the significances were determined by non-parametric one-way ANOVA (*** *p* < 0.001).

The enhanced antibacterial activity of the COL/NIC-NEs and COL/NIC-NEGs delivery system may be due to the enhanced interaction of the encapsulated drugs with the bacterial cell membrane (40). To verify whether the enhanced antimicrobial activity is related to cell membrane interactions, we performed fluorescent probe assays. First, we investigated the changes in bacterial membrane biochemical parameters treated with the COL/NIC-NEs and COL/NIC-NEGs. NPN and PI were used to evaluate bacterial outer membrane permeability and bacterial cell membrane integrity, respectively. DiSC3 (5) and BCECF-AM were used to detect the electric potential (Δψ) and transmembrane proton gradient (ΔpH) (41). We found that the COL/NIC-NEs and COL/NIC-NEGs increased outer membrane permeability, and whole membrane permeability, and led to the dissipation of PMF by disrupting Δψ and ΔpH. In contrast, free COL did not induce significant changes in the fluorescence intensity of NPN, possibly attributed to the weakened interaction between modified lipopolysaccharide and COL in COL-resistant bacteria (*mcr-1* positive). In addition, the fluorescence intensity of NPN and PI was significantly enhanced in the groups of COL/NIC-NEs and COL/NIC-NEGs compared to the free COL and NIC groups. This suggests that the COL/NIC-NEs and COL/NIC-NEGs can improve the fusion with cell membranes, thereby enhancing cell membrane permeability (Figures 6A,B). The PMF is that it consists of two components including electric potential (Δψ) and transmembrane proton gradient (ΔpH), which continually work together to maintain homeostasis (42). We measured the fluorescence intensity of the fluorescent probes DiSC3 (5) and BCECF-AM in the tested strains, respectively. The fluorescence intensity of the COL/NIC-NEs and COL/NIC-NEGs is significantly enhanced, indicating that the dissipation of PMF is accelerated by destroying the membrane potential and ΔpH (Figures 6C,D).

It has been shown that NIC can act as an efflux pump inhibitor to reverse the resistance of *Klebsiella pneumoniae* and *Acinetobacter baumannii* (21), thus promoting the retention of COL in the cell and enhancing their antibacterial activity. In order to investigate whether NIC inhibits drug efflux and thus improves the antibacterial activity of NIC against COL-resistant bacteria, the efflux effect of COL-resistant *Salmonella* on EtBr was studied. Compared to the control group without any treatment, both free NIC and free combination of NIC and COL increased fluorescence intensity, indicating an inhibitory effect on the efflux pump of EtBr (Figure 6E). More importantly, the fluorescence intensity of the COL/NIC-NEs and COL/NIC-NEGs was significantly increased, suggesting that they can enhance antimicrobial activity by inhibiting the activity of the efflux pump.

Studies have reported that various antibiotics can affect different cellular targets by causing a reactive oxygen species (ROS)-mediated disturbance in metabolism and respiration (43, 44). ROS is a common mechanism of antibacterial agents, and it can promote cell oxidative damage by increasing ROS generation. Therefore, we sought to detect changes in ROS levels in different drug combinations. The results indicated that the free NIC and COL combination group led to increased ROS levels better than the COL monotherapy group. Interestingly, the ROS levels significantly increased in the COL/NIC-NEs and COL/NIC-NEGs compared with the free NIC and COL combination group (Figure 6F). Thus, the above results showed that oxidative stress of bacteria cells exposure to the COL/NIC-NEs and COL/NIC-NEGs led to increasing intracellular ROS levels.

### 3.10 Evaluation of the antibacterial activity of COL/NIC-NEs and COL/NIC-NEGs *in vivo*

**Figure 7:**
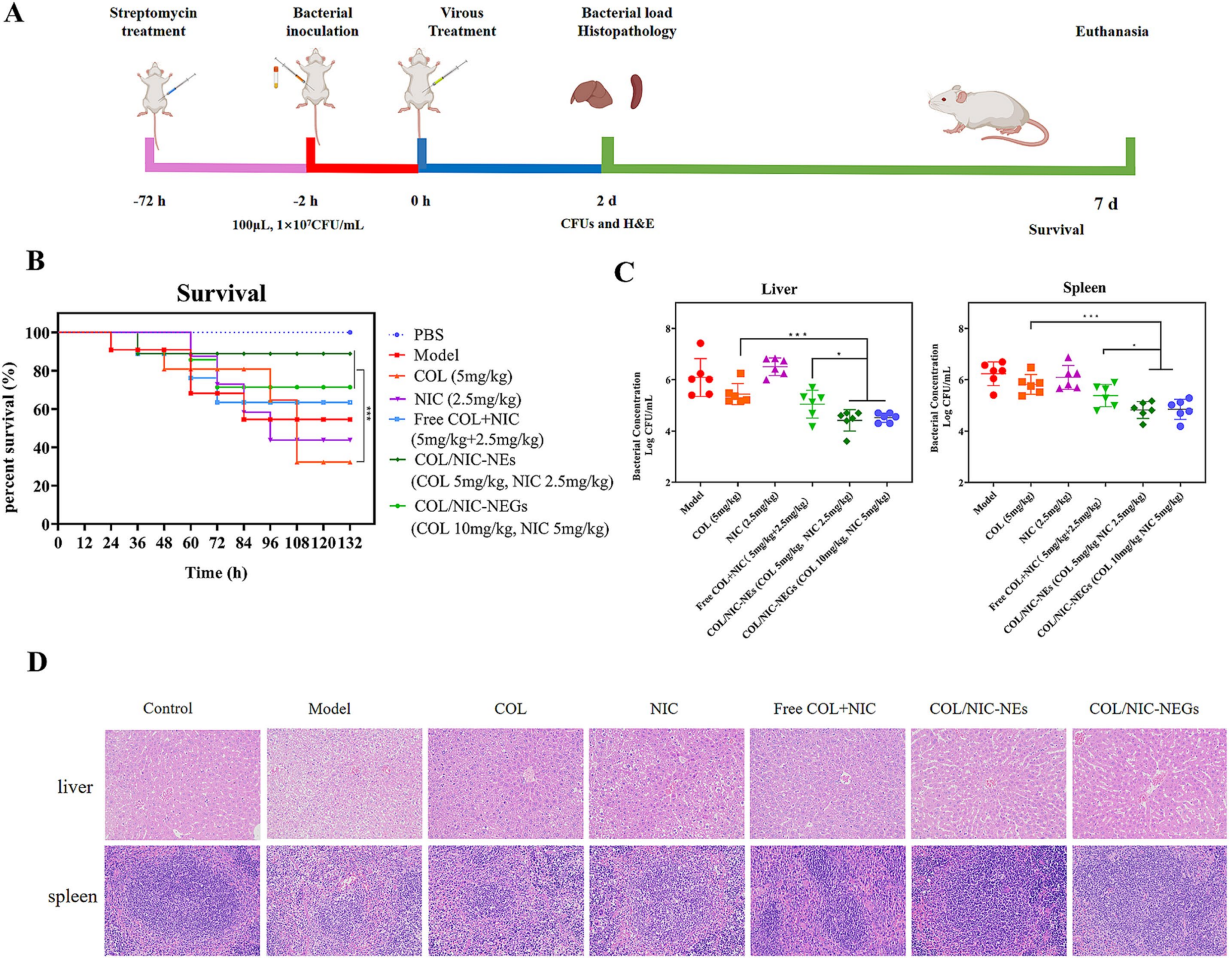
Therapeutic effects of COL/NIC-NEs and COL/NIC-NEGs in a mouse intraperitoneal infection model of colistin-resistant *Salmonella*. **(A)** Scheme of mouse infection model monitoring, including survival rate, time of treatment, bacterial loading, and histopathological observation. The treatment of COL/NIC-NEs and COL/NIC-NEGs improved the survival rate of mice **(B)** and reduced the *Salmonella* bacterial load in the liver and spleen of a mouse abdominal infection model **(C)**. **(D)** H&E staining of the liver and spleen. *p*-values were determined by the Mann–Whitney U-test. *p*-values (** *p* < 0.01 and **** *p* < 0.0001) are reported. Data are presented as mean ± SD.

The gastrointestinal tract serves as a critical site for the colonization of *mcr-1*-positive *Salmonella*, and these bacteria have been isolated from patients with intra-abdominal infections. To investigate the synergistic antibacterial activity and therapeutic potential of COL/NIC-NEs and COL/NIC-NEGs *in vivo*, a mouse model of abdominal infection was established through the injection of clinically isolated COL-resistant *Salmonella* (Figure 7A).

To that end, we tested the *in vivo* efficacy of this combination in mouse infection models with *Salmonella* SH134. As shown in Figure 7B, treatment with alone NIC and COL did not exert adequate protection in the infected mice. Excitingly, administration of COL/NIC-NEs and COL/NIC-NEGs significantly improved the survival rate (*p* < 0.001), with survival increasing from 37.5% (COL) to 87.5 and 75%, respectively. In particular, when compared to the free COL+NIC group, COL/NIC-NEs and COL/NIC-NEGs were able to further boost the survival rates of mice at an equivalent dose, thus further corroborating that COL/NIC-NEs and COL/NIC-NEGs can potentiate the therapeutic efficacy of COL and NIC. Similarly, the expected efficacy of combination therapy *in vivo* was also observed in the bacterial load in the liver and spleen of mice (Figure 7C). More specifically, the combinational therapy with COL/NIC-NEs and COL/NIC-NEGs exhibited approximately a 2-log_10_ reduction in colony-forming units (CFUs) compared to treatment with COL and NIC alone.

Finally, the therapeutic effectiveness of this combination was evaluated through histopathological examination of liver and spleen tissues (Figure 7D). HE staining demonstrated prominent cell necrosis in hepatocytes of the model group and in the groups treated exclusively with COL and NIC, respectively. However, a protective effect on the liver was observed in the treatment groups of COL/NIC-NEs and COL/NIC-NEGs. In the spleen sections, the demarcation between the white and red pulp was unclear in the model group, the NIC group, and the COL group. In particular, compared to the model group, the NIC group, and the COL group, the spleens of both the COL/NIC-NEs and COL/NIC-NEGs groups exhibited normal histological features. Overall, these findings indicated that COL/NIC-NEs and COL/NIC-NEGs significantly safeguarded mice against COL-resistant *Salmonella* infection.

## 4 Discussion

Infectious diseases caused by MDR Gram-negative bacteria have posed a significant public health challenge due to the scarcity of effective treatments. Given the limited approval of new antibacterial agents, there is an urgent need to develop drug carrier formulations capable of enhancing the activities of already approved antibiotics. This study focuses on the development of COL/NIC-NEs and COL/NIC-NEGs to achieve the co-delivery of NIC and COL, improve the solubility of NIC, enhance stability, and potentiate its synergistic antibacterial activity. Although the literature has reported that this drug combination of NIC enhances the antibacterial activity of COL against *Klebsiella pneumoniae*, *Acinetobacter baumannii*, and *Escherichia coli* (17, 21), their clinical usage has been significantly hindered by poor solubility, low bioavailability, lack of targeting, and other shortcomings.

Nanotechnology can improve the solubility of insoluble drugs, and its inherent good properties promote antibacterial activity against many microorganisms, including bacteria, viruses, and fungi (24). Recently, advanced drug delivery systems have made considerable progress in the treatment of bacterial infections based on nanoemulsion (52), nanoemulsion gel (45), polymeric micelles (46), liposomes (40), and nanoparticles (47). For example, a liposomal delivery system loaded with curcumin and colistin exhibits the potential to overcome colistin resistance and may be used to treat colistin-resistant bacterial infections (40). Study by Sheng Qiu Shuang has elucidated a novel approach utilizing naringenin microspheres to restore the efficacy of colistin against colistin-resistant Gram-negative bacteria. This finding presents a potentially effective strategy for the development of novel therapeutic candidates targeting multidrug-resistant (MDR) Gram-negative bacterial infections (48). In this study, a self-emulsifying nanoemulsions and nanoemulsion gels drug delivery system was prepared to simultaneously encapsulate hydrophilic COL and lipophilic NIC. Moreover, these COL/NIC-NEs and COL/NIC-NEGs can encapsulate hydrophobic drug NIC into the hydrophobic inner core, increasing the solubility of NIC. Using preliminary solubility testing and pseudo-ternary phase diagrams, EO, ethanol, and EL were selected as the main carriers for nanoemulsions. A good surfactant must consider the solubility of the drug, the hydrophilic lithium balance (HLB), and toxicity to the body (49). We found that EL has the highest solubility and good compatibility with NIC, EL was used as a surfactant for nanoemulsions. Ethanol is non-irritating, has gentle properties, has no toxicity to the human body, and is the most common and readily available reagent, and it was chosen to be the co-surfactant (50). The COL/NIC-NEs with small particle size and stable morphology, thus, validate the success of our preparation of nanoemulsions. In this study, thermal sensitivity gel was selected as the appropriate matrix for dispersing nanoemulsion, because it has good biocompatibility and temperature sensitivity (51). P407 and P188 with thermal sensitivity are used as gelling agents to prepare thermal-sensitive gel from nanoemulsion. Our results show that the COL/NIC-NEGs composite system maintained the temperature-sensitive property with a transparent yellowish solution state at 2–8°C and a gel state at 36–38°C. In addition, the *in vitro* release experiment proved the slow-release effect of the COL/NIC-NGEs.

Encouragingly, the antibacterial activity *in vivo* and *in vitro* showed that the COL/NIC-NEs and COL/NIC-NEGs had better therapeutic effects. Compared with free COL, the antibacterial activity of the COL/NIC-NEs and COL/NIC-NEGs was 16–128 times higher, and the colonization of *Salmonella* was effectively eliminated *in vivo*, underscoring their potential in tackling drug-resistant bacterial infections. *Salmonella* enteric infection model in mice demonstrated the antibacterial activity and safety of nanoemulsions and nanoemulsion gels. In particular, the COL/NIC-NEGs exhibit a prolonged and sustained-release effect, enabling a reduction in administration frequency while achieving the dual objectives of dosage reduction and efficacy enhancement. Furthermore, the remarkable sustained-release capability of the COL/NIC-NEGs has been conclusively demonstrated *in vivo*. The primary antimicrobial mechanism of the COL/NIC-NEs and COL/NIC-NEGs relies on ability of nanoemulsions to fuse with bacterial cell membranes and increase their permeability, leading to the leakage of cellular contents and bacterial death, consonant with mechanisms underpinning some existing nanodelivery system (40). Furthermore, the COL/NIC-NEs and COL/NIC-NEGs can inhibit drug efflux and promote bacterial oxidative damage, leading to bacterial death, which are also important mechanisms for enhancing antibacterial activity. In addition, further research is needed on the pathways involved in inflammation caused by bacterial infections, in order to provide direction for subsequent research and new treatment strategies, such as COL/NIC NE and COL/NIC-NEG as adjunctive drugs for treating drug-resistant bacterial wound infections. In summary, the COL/NIC-NEs and COL/NIC-NEGs, with simplified preparation process, good stability, enhanced antibacterial activity of drugs, and improved efficacy, can be used as a potential method to treat drug-resistant bacterial infections.

## 5 Conclusion

**Figure 8:**
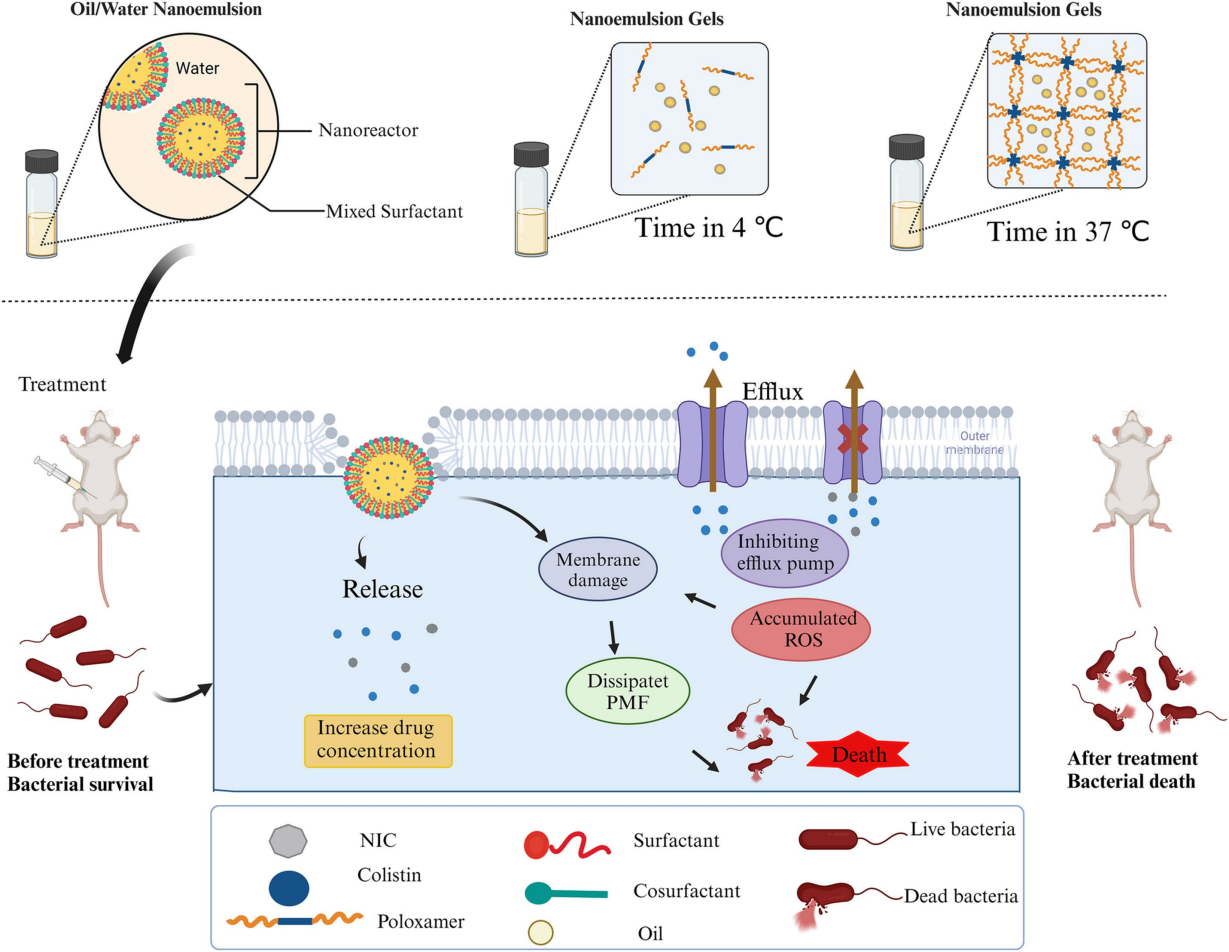
Schematic illustration of the self-nanoemulsifying co-delivery system encapsulating both niclosamide and colistin and a treatment schedule for treating mouse infections induced by colistin-resistant *Salmonella* using COL/NIC-NEs and COL/NIC-NEGs. Created with Biorender (biorender.com).

In this study, we confirmed that the NIC reversed the resistance phenotype of COL-resistant *Salmonella.* We developed a self-nanoemulsifying co-delivery system encapsulating both NIC and COL, termed as COL/NIC-NEs and COL/NIC-NEGs. Compared with free COL, this co-delivery system significantly enhances its antibacterial effect against clinically isolated COL-resistant *Salmonella*. The enhanced antibacterial activity of COL/NIC-NEs and COL/NIC-NEGs was attributed to multiple mechanisms, including increased cell membrane damage, disruption of the proton motive force (PMF), inhibition of multidrug efflux pumps, and potentiation of oxidative damage. Furthermore, the COL/NIC-NEs and COL/NIC-NEGs achieve continuous antibacterial effects through sustained drug release. More importantly, the COL/NIC-NEs and COL/NIC-NEGs significantly reduced COL-resistant *Salmonella* load in the liver and spleen and improved the survival rate of COL-resistant *Salmonella*-infected mice in a mouse infection model (Figure 8). Thus, the drug delivery system (COL/NIC-NEs and COL/NIC-NEGs) provides a promising strategy for clearing bacterial infections, which has potential value in clinical application.

## Data Availability

The original contributions presented in the study are included in the article/Supplementary material, further inquiries can be directed to the corresponding authors.
