# Supplementary material for: Colistin–niclosamide-loaded nanoemulsions and nanoemulsion gels for effective therapy of colistin-resistant Salmonella infections
Source: Front Vet Sci. 2024 Oct 23;11:1492543. doi: 10.3389/fvets.2024.1492543 (PMC11539104; doi:10.3389/fvets.2024.1492543)
Supplement: Supplementary file 1 [file Data_Sheet_1.docx]

**SUPPLEMENTARY MATERIAL**

**Colistin-niclosamide-loaded nanoemulsion and nanoemulsion gels for effective therapy of colistin-resistant Salmonella infections**

Junkai Zhang^1†^, Xilong Wang^1†^, Yanling Gao^2†^, Ruiyun Wang^1^, Shuaihua Li^1^, Xingwei Luo^1^, Xiaodie Cui^1^, Gongzheng Hu ^1^* and Yajun Zhai^1^*

^1^ College of Veterinary Medicine, Henan Agricultural University, 450002, Zhengzhou, China

^2^ Henan Vocational College of Agriculture, 451450, Zhengzhou, China

^†^ The authors contributed equally as the first author

* Corresponding author

*Correspondence: Yajun Zhai : [zyj90518@126.com;](mailto:zyj90518@126.com;) Gongzheng Hu : Yaolilab@126.com;


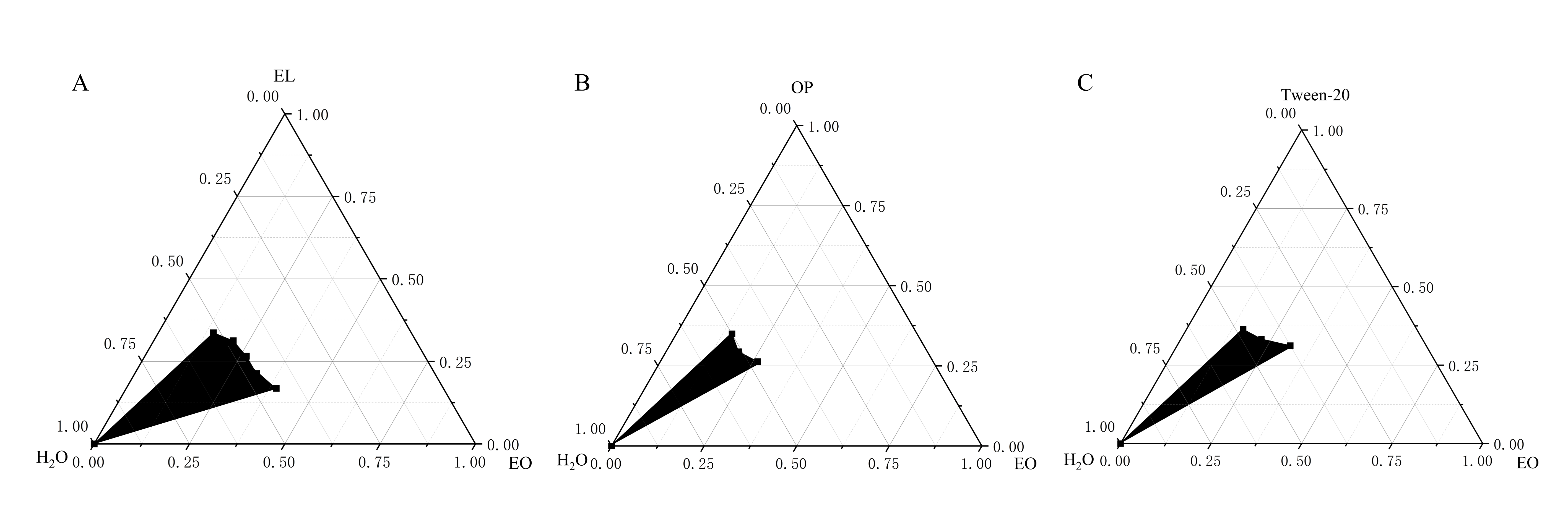


**FIGURE S1** Pseudoternary phase diagrams of different surfactant indicating the nanoemulsion region (A) EL, (B) OP and (C) Tween-20.


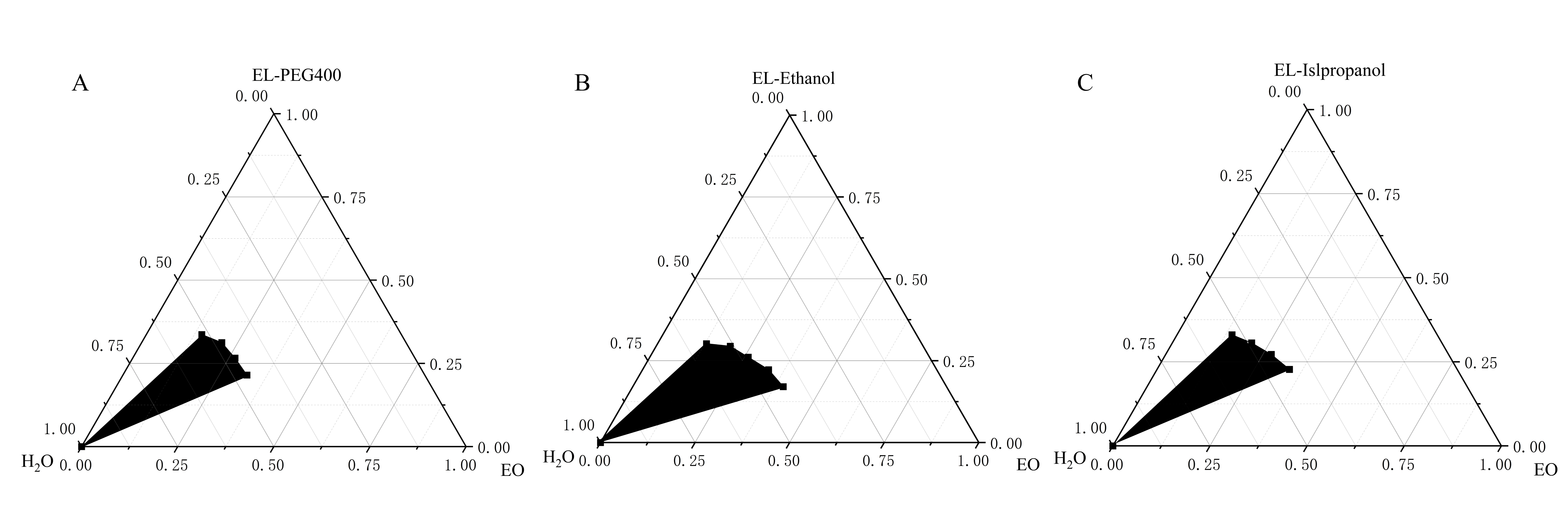


**FIGURE S2** Pseudoternary phase diagrams of different co surfactant indicating the nanoemulsion region (A) PEG400, (B) Ethanol and (C) Islpropanol.

**
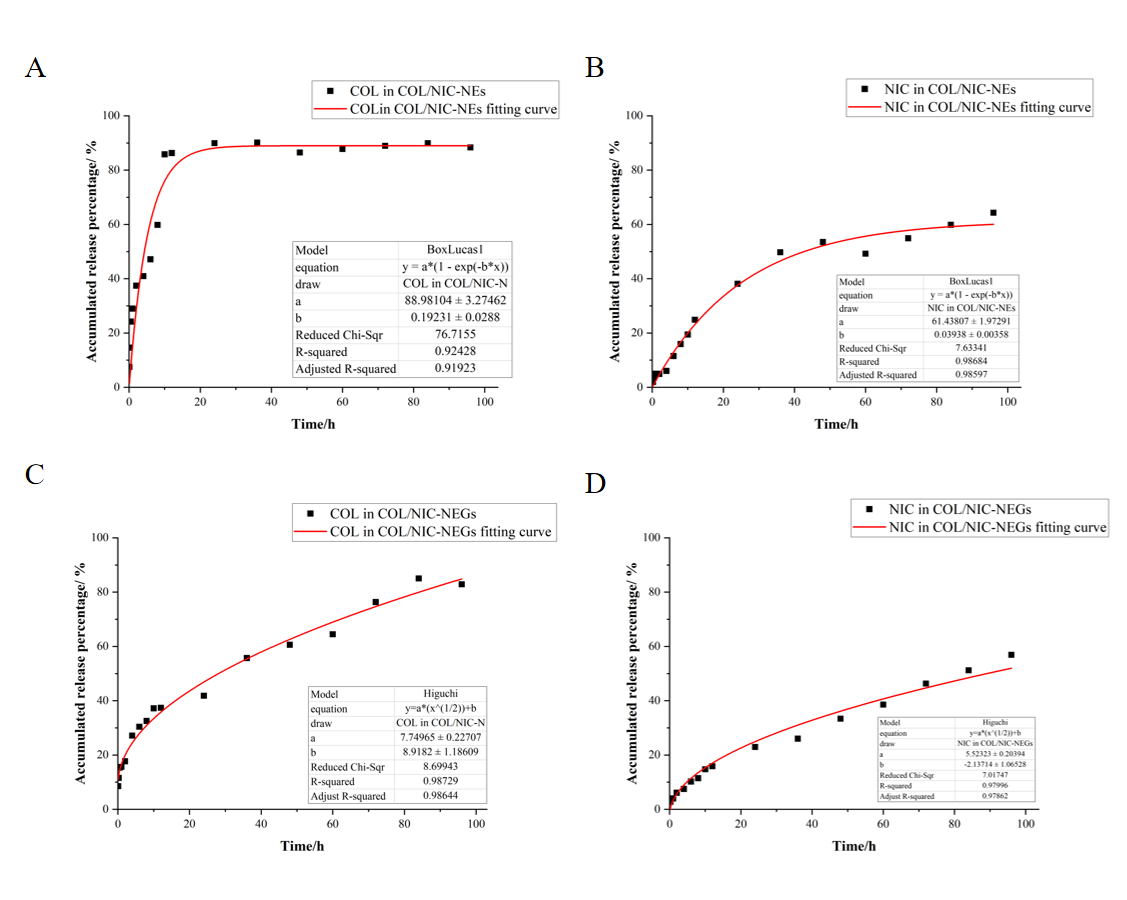
**

**FIGURE S3** COL/NIC-NEs, COL/NIC-NEGS in vitro release fitting curves and fitting formulas.

(A, B) Fitting curves and equations for in vitro release of COL and NIC in COL/NIC-NEs, (C, D) Fitting curves and equations for in vitro release of COL and NIC in COL/NIC-NEGs.


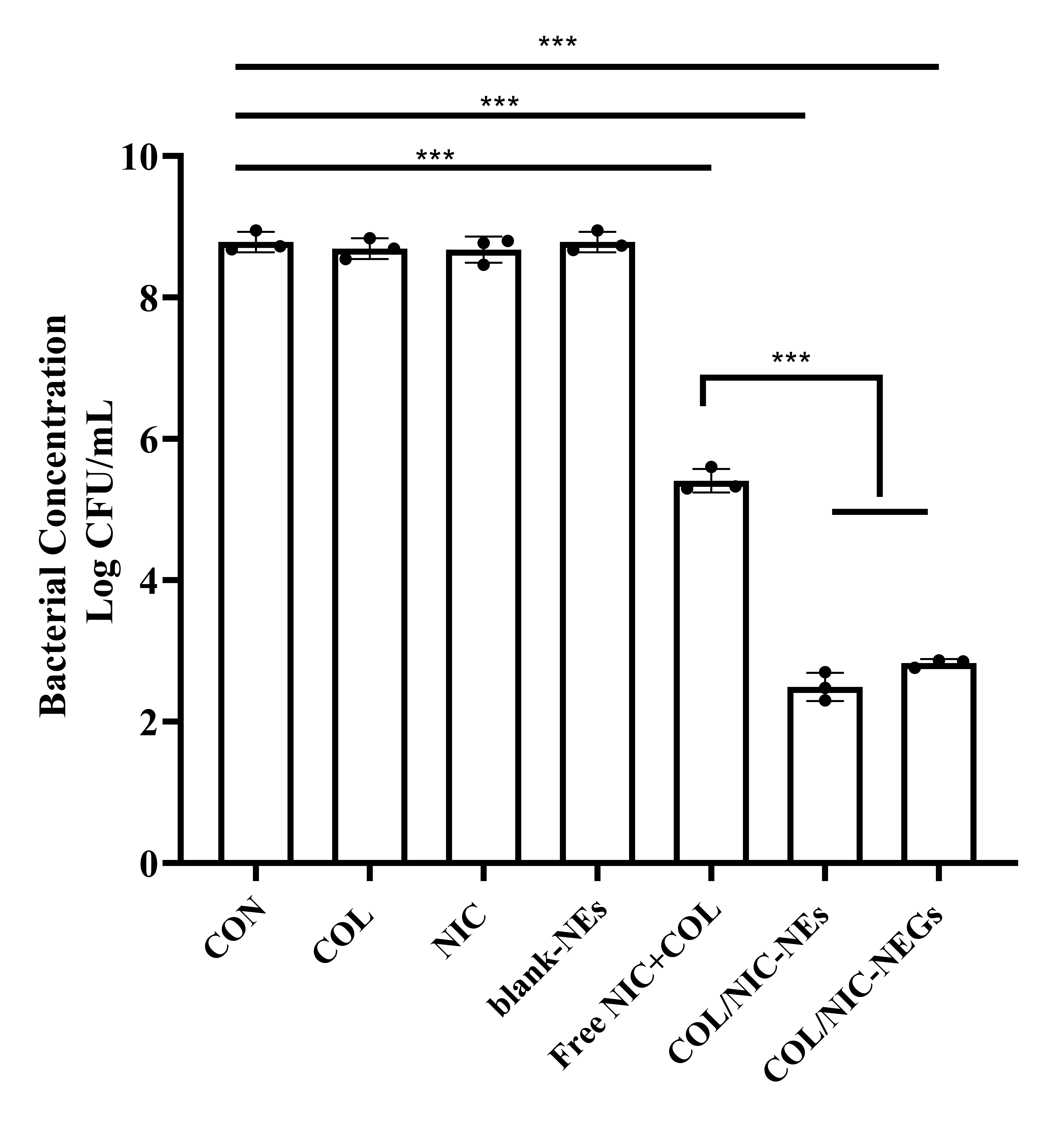


**FIGURE S4** Killing efficacy of niclosamide (NIC, 4 μg/mL), colistin (COL, 8 μg/mL), free combination of niclosamide (4 μg/mL) and colistin (8 μg/mL), COL/NIC-NEs and COL/NIC-NEGs ( containing 4 μg/mL niclosamide and 8 μg/mL colistin) after incubating with *Salmonella* SH134 for 24 h (n = 3, dot line representing limit of detection: 100 CFU/mL).

**TABLE S1** Different NIC and COL loaded NEs formulations.

| Formulation | EO  (g) | EL  (g) | ethyl alcohol (g) | Colistin  (mg) | Niclosamide  (mg) | Size  (nm) | PDI  (%) |
| --- | --- | --- | --- | --- | --- | --- | --- |
| NE1 | 1.5 | 2.1 | 1.4 | 40 | 20 | 107.1±0.61 | 0.18±0.03 |
| NE2 | 2.5 | 1.5 | 1 | 40 | 20 | 152±4.58 | 0.73±0.03 |
| NE3 | 4.5 | 0.3 | 0.2 | 40 | 20 | 158.67±3.1 | 0.43±0.04 |
| NE4 | 3.5 | 0.9 | 0.6 | 40 | 20 | 192.13±13.7 | 0.97±0.06 |
| NE5 | 1 | 2.4 | 1.6 | 40 | 20 | 94.03±11.7 | 0.31±0.08 |
| NE6 | 0.5 | 2.7 | 1.8 | 40 | 20 | 19.86±2.21 | 0.103 ± 0.01 |

**TABLE S2** MIC values of colistin, niclosamide, colistin in COL/NIC-NEs and COL/NIC-NEGs against different bacterial strains. The initial concentration of colistin, colistin in COL/NIC-NEs and COL/NIC-NEGs is 2000 μg/mL, respectively.

| Species | Strain |  | *mcr-1* |  | | MIC value (μg/mL) | | | |
| --- | --- | --- | --- | --- | --- | --- | --- | --- | --- |
|  |  | Source |  | COL | COL in COL/NIC-NEs | | MIC fold  change | COL in COL/NIC-NEGs | MIC fold  change |
| *Salmonella* | SH134 | Pig | + | 12.5 | 0.39 | | 32 | 0.39 | 32 |
|  | SH30 | Pig | + | 12.5 | 0.39 | | 32 | 0.78 | 16 |
|  | S290 | Pig | + | 25 | 0.195 | | 128 | 0.39 | 64 |
|  | F30 | Pig | + | 25 | 0.195 | | 128 | 0.39 | 64 |
|  | SB05 | Pig | + | 25 | 0.39 | | 64 | 0.39 | 64 |
|  | F108 | Pig | + | 12.5 | 0.39 | | 32 | 0.195 | 64 |
|  | S2a | Chicken | － | 12.5 | 0.195 | | 64 | 0.195 | 64 |
|  | 11R | Human | － | 25 | 0.195 | | 128 | 0.195 | 128 |
|  | S208 | Chicken | － | 6.25 | 0.195 | | 32 | 0.195 | 32 |
|  | SLG | Chicken | － | 25 | 0.195 | | 128 | 0.39 | 64 |
|  | JS | - | － | ＜0.1 | ＜0.1 | |  | ＜0.1 |  |
